# Measures of Dogs' Inhibitory Control Abilities Do Not Correlate across Tasks

**DOI:** 10.3389/fpsyg.2017.00849

**Published:** 2017-05-24

**Authors:** Désirée Brucks, Sarah Marshall-Pescini, Lisa Jessica Wallis, Ludwig Huber, Friederike Range

**Affiliations:** ^1^Comparative Cognition Unit, Messerli Research Institute, University of Veterinary Medicine, Medical University of Vienna, University of ViennaVienna, Austria; ^2^Department of Ethology, Eötvös Loránd UniversityBudapest, Hungary

**Keywords:** inhibitory control, dogs, persistency, delay of gratification, test battery

## Abstract

Inhibitory control, the ability to overcome prepotent but ineffective behaviors, has been studied extensively across species, revealing the involvement of this ability in many different aspects of life. While various different paradigms have been created in order to measure inhibitory control, only a limited number of studies have investigated whether such measurements indeed evaluate the same underlying mechanism, especially in non-human animals. In humans, inhibitory control is a complex construct composed of distinct behavioral processes rather than of a single unified measure. In the current study, we aimed to investigate the validity of inhibitory control paradigms in dogs. Sixty-seven dogs were tested in a battery consisting of frequently used inhibitory control tests. Additionally, dog owners were asked to complete an impulsivity questionnaire about their dog. No correlation of dogs' performance across tasks was found. In order to understand whether there are some underlying behavioral aspects explaining dogs' performance across tests, we performed principle component analyses. Results revealed that three components (persistency, compulsivity and decision speed) explained the variation across tasks. The questionnaire and dogs' individual characteristics (i.e., age and sex) provided only limited information for the derived components. Overall, results suggest that no unique measurement for inhibitory control exists in dogs, but tests rather measure different aspects of this ability. Considering the context-specificity of inhibitory control in dogs and most probably also in other non-human animals, extreme caution is needed when making conclusions about inhibitory control abilities based on a single test.

## Introduction

The ability to stop an immediate but disadvantageous behavior in favor of a more advantageous behavior has been termed “inhibitory control.” While in humans the focus often is on measuring cognitive inhibition, i.e., the ability to regulate low-level actions that are irrelevant to the task (e.g., Stroop task: Stroop, 1935; Stop-Signal task: Verbruggen and Logan, 2008), the focus in non-human animals is generally more on response or *motor inhibition*, i.e., the regulation of prepotent responses (e.g., detour-reaching tasks: Diamond and Gilbert, 1989). Indeed, inhibitory control has been studied in a wide variety of species ranging from bees (Mayack and Naug, 2015), to great apes (e.g., Amici et al., 2008; Vlamings et al., 2010), and of course humans (e.g., Tsukayama et al., 2012). In humans, inhibitory control abilities have been related to many different aspects of life, for example, they have been positively associated with general success in terms of wealth and health (e.g., Moffitt et al., 2011), and negatively associated with addictions (e.g., Smith et al., 2014) and obesity (e.g., Hofmann et al., 2009). Studies in non-human animals have linked enhanced motor inhibition skills to absolute brain volume along with dietary breadth (MacLean et al., 2014) and to more complex social organizations (i.e., fission-fusion dynamics, Amici et al., 2008). In general, inhibitory control appears to be positively associated with problem solving skills, leading many researchers to investigate this link both in the human (e.g., Diamond, 1990; Romer et al., 2009) and non-human literature (dogs: Müller et al., 2016; e.g., chimpanzees: Vlamings et al., 2010; cottontop tamarins: Hauser et al., 2002).

Inhibitory control has been assessed using multiple approaches aimed at measuring different aspects of this ability. Apart from measuring motor inhibition in non-human animal research, *cognitive inhibition* has also been studied using simplified versions of tasks designed for humans. Among those is the frequently used reversal-learning test (e.g., Tapp et al., 2003; Bond et al., 2007; Wobber and Hare, 2009), which assesses cognitive inhibition on the one hand in terms of flexibility in re-learning object-reward contingencies, but on the other hand also inhibition in avoiding the previously rewarded option. Flexibility in reversing the learned reward contingencies seems to be related to social complexity, suggesting that highly social species exhibit greater flexibility allowing them to cope with fluctuations in social context (e.g., Bond et al., 2007) however, also feeding ecology might be a predictor for behavioral flexibility (e.g., Tebbich et al., 2010).

A different approach to measure one distinct aspect of inhibitory control—*self-control (*defined as the capacity to obtain a higher valued outcome through tolerating a certain effort; see Beran, 2015 for a review)—are delay of gratification tasks, in which individuals are required to make a temporal decision between an immediate low-quality reward and a delayed high-quality reward (Mischel et al., 1989). These self-control tasks require individuals to make an active choice between two options, in contrast to inhibitory control tasks where individuals are required to inhibit certain prepotent behaviors without involving an actual choice. In a comparative study by Stevens (2014), the performance of 13 primate species in a delay of gratification task was assessed, revealing that allometric variables (i.e., body mass, brain volume, lifespan, and home range size) predicted waiting success. Accordingly, factors relating to body size seem to be more important in determining self-control abilities than cognitive or social variables among primates.

Finally, a rather different approach to assess inhibitory control is based on self-reported (or in case of non-human animals, caretaker/owner-reported) *questionnaires*. Many different validated questionnaires exist not only for humans (e.g., self-control scale; Tangney et al., 2004), but also for non-human animals (e.g., dog impulsivity assessment scale; Wright et al., 2011).

Surprisingly, studies have found that measures obtained across tasks designed to test the same inhibitory control abilities are not always consistent and consequently a lack of consistency is also found across methodological approaches. In a meta-analysis of inhibitory control studies in humans, Duckworth and Kern (2011) found that inhibition measures were not correlated across methodological approaches (e.g., questionnaires and cognitive inhibition tasks), and especially tasks measuring cognitive inhibition or delay of gratification show a low consistency. Based on this and other studies (e.g., Dougherty et al., 2003; Roberts et al., 2011; Tsukayama et al., 2012), inhibitory control in humans has been proposed to be strongly context dependent. Additionally, it cannot be measured by a single task, and—rather than a unitary mechanism—“inhibitory control” should be considered as a collection of distinct processes (e.g., attention, response inhibition, working memory, task switching, etc.; e.g., Miyake et al., 2000; Friedman and Miyake, 2004). Despite these inconsistencies in measurements of human inhibition skills, at least two studies have assessed *motor inhibition* across species using multiple tests. For example, MacLean et al. (2014) collected data on inhibitory control across 36 species of mammals and birds in two motor inhibition tasks (i.e., a detour-reaching and an A-not-B task) to analyze which factors (e.g., sociality, dietary breadth, brain size) explain the motor inhibition in the species. This study reported that the two inhibition tasks were indeed strongly correlated. A second study by Amici et al. (2008) tested eight primate species in a battery of four motor inhibition tasks (i.e., A-not-B task, middle cup task, detour-reaching task, and swing door task) and one delay of gratification task. Unfortunately, this study did not validate whether individual performance was correlated across tasks. The authors only tested for species differences within each task, making claims based on the species' overall performance independent of testing for internal consistency.

Whereas in the human literature some attention has been given to the difficulties in measuring inhibitory control, only a handful of studies have addressed this issue in the non-human animal literature. Bray et al. (2013) tested the consistency of inhibition measures in dogs using a test battery (A-not-B, detour-reaching and a social inhibition task), and found that dogs' performance was not correlated across tests. Likewise, two other canine studies found no correlation in performance across different inhibition tests (Marshall-Pescini et al., 2015: detour and detour-reaching task; Müller et al., 2016: middle cup, leash detour and wait-for-treat task). Considering the complexity of inhibitory control and the resulting inconsistencies across tasks, it remains questionable whether any single measure can be considered a complete evaluation of an individual's inhibition skills. Consequently, if inhibitory control is linked to other abilities, like problem solving, it can result in a positive or negative association depending on the chosen inhibition task and the aspect of inhibition the task measures. Thus, in order to draw valid conclusions about the role of inhibitory control in more complex behaviors like cooperation (e.g., Lakshminaryanan and Santos, 2009; Giannotta et al., 2011) or behavioral flexibility (Amici et al., 2008), and/or assess the potential factors affecting inhibitory or self-control over multiple taxa (MacLean et al., 2014; Stevens, 2014), it is necessary to ascertain whether specific tests are indeed capturing the inhibitory capacity of a species. To overcome this issue, animals' inhibitory control abilities need to be assessed using a variety of different measures, although most of the studies to date utilized only a single inhibition measure (e.g., single test: Miller et al., 2010; Bray et al., 2015; e.g., questionnaire: Fadel et al., 2016).

A first step in understanding inhibitory control within a given species is to test whether a categorization into motor inhibition, cognitive inhibition and self-control is universally applicable, and secondly to validate that the different inhibition tests indeed capture these specific aspects of inhibition (e.g., whether detour-reaching actually tests for motor inhibition). Without this understanding of a species' whole inhibition capacity, no claims can be made about the involvement of inhibitory control in other behaviors. Dogs represent an interesting model species to investigate the complexity of inhibitory control since they have already been tested in a variety of inhibitory control tasks, are available in great numbers, and can easily be trained to perform different tests. Furthermore, a delay of gratification test has been recently successfully developed with this species (Brucks et al., 2017) and a validated questionnaire is already available (Wright et al., 2011), allowing us to include these two measures of inhibition.

In this study we present a different approach to the study of inhibitory control by using multiple tests, aimed at assessing different aspects of inhibition (i.e., motor inhibition, cognitive inhibition/flexibility, self-control) within individuals. Our aim was 2-fold; firstly, we wanted to investigate whether dogs' inhibitory control performance was consistent across different contexts by correlating the conventionally used measures of inhibitory control. Secondly, if dogs' performance was not stable across tasks, we were interested in investigating what underlying mechanisms these tasks actually measure. Accordingly, we tested dogs in five different tests based on the previous literature and their adaptability for use on dogs. We included three motor inhibition tests: a detour reaching task, a middle cup test and a new designed buzzer test.

The detour-reaching task, dubbed “the box test,” has previously been used with children (Diamond and Gilbert, 1989; Lockman and Adams, 2001) but also with cotton-top tamarins (Santos et al., 1999), rhesus monkeys (Diamond, 1990), and squirrel monkeys (Parker et al., 2005). The underlying principle of this test lies in the fact that individuals need to inhibit reaching for a visible food reward directly and instead must make a detour to gain access to it, hence requiring inhibition of salient action tendencies. The “box test” is similar to the previously utilized cylinder task (e.g., MacLean et al., 2014; Marshall-Pescini et al., 2015), as both consist of a training phase (i.e., opaque, no reward visible), and both introduce a novel task context (i.e., transparent barrier, hence visible food reward). However, in contrast to the cylinder task, the test also allows different levels of difficulty (i.e., food distance from the opening), which is absent in the cylinder task.

The second motor inhibition task was the “middle cup test,” previously used with different primate species (Call, 2001; Amici et al., 2008). This task was originally designed to test for object permanence, but was shown to elicit inhibition problems. In this test, subjects are presented with three transparent cups aligned in a row under which two pieces of rewards are visibly hidden. Two cups can be chosen in each trial. One cup remains empty and the subject should avoid choosing it. Inhibitory problems are observed when subjects are unable to avoid the empty cup when the rewards were hidden under non-adjacent cups. The middle cup task was included in this study because it adds another context for testing response inhibition. In particular, the middle cup test entails a choice component (contrary to the box task), while additionally enhancing the saliency of the food rewards in visibly presenting them (contrary to the reversal learning task).

The third task to assess motor inhibition was a setup we designed that involved different aspects. In this test, dubbed the “buzzer test,” dogs were first trained to open an opaque box by pressing a buzzer placed next to the box. In the test phase, they were presented with the food reward, which was placed inside a transparent box, and the buzzer was now located behind the dog and in the opposite direction of the box. Consequently, to obtain the food reward in the box, dogs needed to inhibit directly approaching and manipulating the box, and instead turn away from it to press the buzzer placed in the opposite direction. We designed this test because it adds a new experimental component to study of response inhibition (i.e., reaching for the reward directly, similarly to the detour-reaching task), while also involving a learned component, since dogs needed to remember a previously learned behavior (i.e., pressing the buzzer) despite the temptation of visible food.

To assess aspects of “self-control” (e.g., see Beran, 2015 for a review) similarly to studies with other species (e.g., children: Mischel et al., 1989; primates: Stevens, 2014; birds: Dufour et al., 2012; Auersperg et al., 2013, and also dogs: Leonardi et al., 2011), we presented dogs with a delay of gratification test. Typically, in delay of gratification tests, individuals are given the choice between two reward options of different quality. In order to obtain the high quality reward, individuals need to resist consuming the immediately available low quality reward for a certain delay. Consequently, in delay of gratification tasks individuals must make a decision based on the effort (i.e., delay) that is necessary to obtain the better reward. Eventually, individuals reach an indifference point at which it no longer pays off to wait for the better reward because it becomes too costly (requires waiting too much time). For example, chimpanzees wait for a better reward for several minutes (e.g., Beran, 2002; Dufour et al., 2007), while rats reach their indifference point at only several seconds (Reynolds et al., 2002). Accordingly, in the delay of gratification test, dogs were given the choice between an immediately available low quality reward and a visible but inaccessible high quality reward, which was only made available after a certain delay.

Finally, to assess aspects of dogs' flexibility and cognitive inhibition, a reversal-learning test was included in the battery. Reversal learning tests are object discrimination tasks utilizing two stimuli that change in their reward contingencies after the initial discrimination learning. More specifically, first, individuals need to learn an association between a particular object and a food reward (S+), while the second object, the negative object is never paired with a food reward (S−). Once the individuals reach a learning criterion (i.e., successfully choosing S+), the reward contingencies are reversed in a way that S+ is no longer rewarded, while S− is rewarded. Apart from giving information about learning capacities, this test also involves an inhibitory control component, since the individuals need to inhibit choosing S+ after the reward contingencies are reversed in order to succeed (Rumbaugh, 1971). Reversal learning tasks have frequently been used in the literature across a range of different species (e.g., pigeons: Laude et al., 2016; dogs: Tapp et al., 2003; mice: Pattij et al., 2003; tamarins: Hauser et al., 2002; corvids: Bond et al., 2007; chimpanzees: Wobber and Hare, 2009), not only to assess learning capacities but also to test for behavioral flexibility and inhibitory control. Here we tested dogs in a simple reversal-learning test with two objects differing both in color and in shape.

To investigate whether dogs' inhibitory control performance was consistent across different contexts, we correlated the animals' performance across tasks. Additionally, in order to examine the underlying mechanisms these tasks actually measured, we subsequently performed principle component analyses (PCA) on all variables obtained from the tests. If a general measure of inhibitory control exists in dogs, we would expect a positive correlation across test performance, and variables to group together on one factor, thereby showing that these tests indeed measure the same underlying process. Alternatively, if inhibitory control is context specific, as suggested by some studies (Bray et al., 2013; e.g., Tsukayama et al., 2012), we would expect no correlation between test performances, or potentially only between tests using a similar approach (e.g., motor inhibition tasks). In this case, the PCA may result in multiple groupings of behaviors, which would help to outline what underlying processes are being measured in such tests. In addition, we investigated whether individual characteristics as well as the scores obtained from the validated questionnaire (Wright et al., 2011), can explain the individual variation of performance in the various tests.

## Methods

We tested 67 dogs (mean age: 5.95 ± 0.36 years, 33F/34M; see Table S1 for details) in a test battery consisting of five different tasks all aiming at measuring inhibitory control. Our test battery included two motor inhibition tests (box test, middle cup test), one behavioral flexibility/cognitive inhibition test (reversal learning test), a delay of gratification test and one newly developed test assessing motor inhibition in the context of a newly learned behavior (buzzer test). Furthermore, an owner-reported questionnaire was carried out. Some dogs did not succeed in all of the tests due to fear of the apparatus or a lack of motivation (35 dogs completed all 5 tests, 23 dogs completed 4 tests, 5 dogs completed 3 tests and 4 dogs completed only 2 tests; see Table S1 for details). Not all dogs were tested in the delay of gratification test due to time restraints (i.e., 37 dogs completed the test, 15 dogs already participated in the delay of gratification test before, see Brucks et al., 2017, and 22 dogs were tested in the current study). All tests were conducted between June 2015 and April 2016 in an empty test room (7 × 6 m) at the Clever Dog Lab. Dogs were allowed to explore the room for 2 min at the beginning of each test day. Owners were required to sign a consent form prior to participation and ethical approval was obtained by the ethical commission of the University of Veterinary Medicine Vienna (Approval number: 10/12/97/2013).

### Questionnaire

Dog owners were asked to fill in a questionnaire about their dogs' impulsivity in daily situations. The Dog Impulsivity Assessment Scale (DIAS) is a validated questionnaire consisting of 18 questions, which can be rated on a 5-point Likert-scale (Wright et al., 2011), which we translated into German. One overall impulsivity score (DIAS; mean ± SD = 0.53 ± 0.09) as well as scores for behavioral regulation (mean ± SD = 0.47 ± 0.14), aggression and response to novelty (mean ± SD = 0.40 ± 0.13), and responsiveness (mean ± SD = 0.73 ± 0.12), were calculated from the questionnaire (see Table S1 for individual scores), following the procedure of Wright et al. (2011).

### Test procedures

The order of the tests was randomized and counterbalanced across dogs. Only one test was conducted per day, with a maximum of two tests per week. In order to ensure a consistently high food motivation, we used high-quality food rewards for each test (i.e., sausage, with the only exception of dry food for the middle cup test). During each test, the dog owner was present in the room and asked to call the dog back and release it during each trial. Owners, who sat behind the dogs, were instructed to remain passive (i.e., no talking to the dog or showing gestures to enhance the dog's performance), and to follow the experimenter's instructions for each test.

### Box test

**Figure 1:**
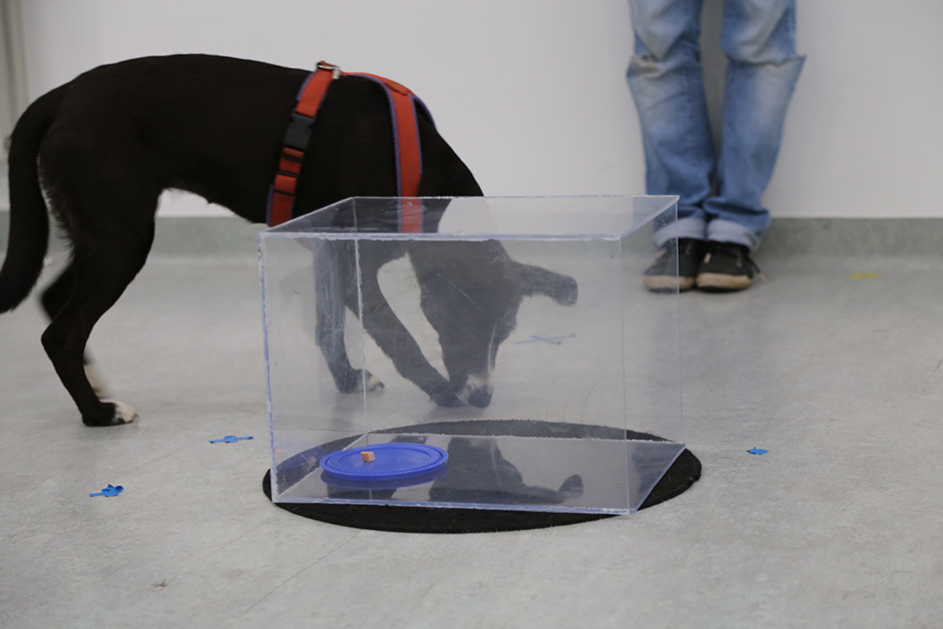
**Setup in the box test. The reward is positioned deep in the box on a lid and the box is open on the right side**. The experimenter stands behind the box.

In this test, dogs were required to retrieve a piece of sausage from inside a Plexiglas-box, which was open only on one side (Figure 1). The dogs consequently needed to inhibit reaching for the reward directly but instead were required to look for the open side of the box (left, right or back) to gain access, hence measuring the dogs' motor inhibition abilities. Moreover, we put the reward either at the center of the box (center condition) or deeply inside the box (deep condition). We adapted this test for dogs following the procedures outlined in Hauser (1999), Lockman and Adams (2001), and Parker et al. (2005).

#### Setup

We used a rectangular shaped Plexiglas box (40 cm long × 30 cm wide × 30 cm height) with one of the smaller sides of the box open. The box was placed on a circular pad in order to prevent it from moving when pushed (see Figure 1). We used pieces of sausage (2 cm diameter) as rewards, which were placed on a blue plastic lid in order to enhance visibility. The owner sat on a chair 2 m away from the box and was instructed to remain silent and static during each trial. The owner released the dog from this position by removing the hand from the collar.

#### Pre-training

In order to facilitate the association between lid and reward, dogs were first exposed to two warm-up trials in which they could retrieve a reward from the lid. The experimenter showed the dog a piece of sausage, placed it on the lid and placed the lid on the floor 2 m distant from the dog. The dog was then released by the owner and allowed to eat the reward. After the owner called the dog back, the next trial started.

#### Procedure

The test started with a *training phase* in which dogs were presented with an opaque box. The experimenter showed the dog a treat, placed it on a lid and put the lid inside the box. As soon as the experimenter stepped back 1.5 m from the box and looked down, the dog was released by the owner and allowed to retrieve the reward. After the dog ate the reward, it was called back again and the next trial started after a 30 s break. Six training trials were conducted, alternating the location of the open side of the box (i.e., left, right, and back) in-between trials. The positioning of the open side was randomized and counterbalanced across training trials.

Directly following the training, the *test trials* started. The box was made transparent by removing the fabric cover and the dog was no longer allowed to witness the baiting. As before, the owner restrained the dog during baiting. A second experimenter placed a curtain in-between the dog and the box, while the first experimenter placed the baited lid inside the box. After the baiting was completed, the curtain was removed, while the experimenter stepped back and looked down. This was the signal for the owner to release the dog. The trial finished either when the dog ate the reward or when 30 s were over. If a dog was not successful in finding the reward, the dog was called back by the owner. Six test trials were conducted. Again, the open side (i.e., left, right, and back) was alternated randomly between trials and additionally the reward location was changed between a center (15 cm inside of box), and a deep location (30 cm inside of box). Each combination of open side and reward location was tested once.

#### Variables

The main inhibition measure for this test was the frequency of errors (i.e., paw or nose touches to box surface; e.g., Lockman and Adams, 2001). In addition, we analyzed the latency to success (i.e., time from releasing dog until its nose entered the box; max. 30 s) separately for both reward location (deep, center) and the time spent close to the box (within a 1 m radius).

### Middle cup test

**Figure 2:**
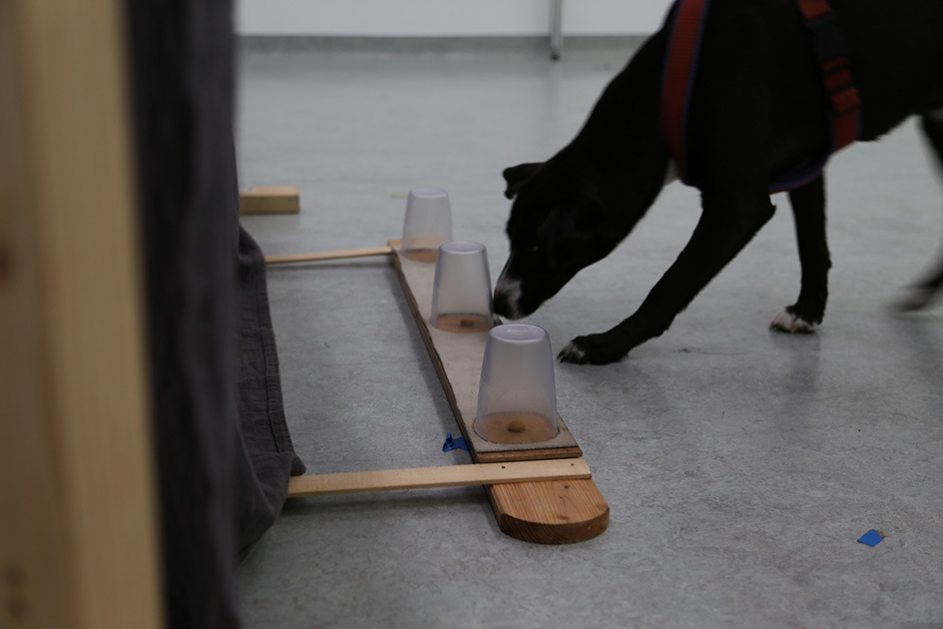
**Setup for middle cup test**. The left and middle cups are baited in this trial.

In this test, dogs were presented with three transparent cups of which only two were baited (see Figure 2). Dogs were allowed to select only two cups. Consequently, in order to obtain both rewards, dogs needed to inhibit knocking over the empty cup.

#### Setup

Three identical transparent plastic cups (11 cm height) were aligned on a wooden board. We followed the procedure by Müller et al. (2016) and deliberately chose transparent cups instead of the opaque cups often used in similar studies (e.g., Amici et al., 2008), in order to reduce the influence of object permanence or memory abilities on dogs' performance, since at least the former is seemingly limited in dogs (e.g., Fiset and Plourde, 2013). The cups were placed at a 30 cm distance from each other in predefined notches. The board was stabilized with two planks of wood. The planks were attached at each end of the board with the latter positioned in front of a curtain (see Figure 2). The experimenter manipulated the cups from behind a curtain in order to reduce the influence of experimenter cues on the dogs. The experimenter could observe the dogs' action via a webcam connected to a laptop behind the curtain. The owner sat on a chair 2 m away from the board and was instructed to release the dog while remaining silent and motionless. Since the rewards were visibly hidden in full view of the dog and additionally, the cups were transparent (hence the dogs could see where the rewards were located), no additional control for scent cuing was implemented.

#### Training

To familiarize dogs with the task (i.e., knocking over cups to gain access to rewards), we conducted two warm-up trials. In warm-up trials, only one cup was baited while the remaining cups were removed. The experimenter showed the dog one piece of dry food (2 cm diameter) and placed this on the wooden board. Then the experimenter placed one over-turned cup over the treat and as soon as she removed her hands behind the curtain, the owner released the dog. When the dog knocked the cup over and ate the reward, the next trial started. If a dog did not knock the cup over, the experimenter helped it by lifting the cup and placing the treat only partially under the cup. If a dog was not able to knock over the cup after 10 repetitions, the dog was excluded from this test (*N* = 2 dogs). The location for the warm-up trials (i.e., left, middle, right) was randomly assigned but counterbalanced between dogs.

#### Test procedure

During the test, the cups were baited according to two conditions: either two adjacent cups were baited (left and middle or right and middle cup; “*control condition”*) or two non-adjacent cups were baited (left and right cup; “*experimental condition”*). Ten trials per condition were conducted (20 trials in total); however, the order of conditions was semi-randomized in a way that no single condition could be tested more than three times in a row. During test trials, all three cups were aligned behind the wooden board, lying with the opening facing toward the dog. The experimenter showed the dogs both treats (one in each hand) and then placed the treats at the same time on the assigned locations. Then, she placed one cup at a time over the assigned notches. The order of placing the cups was randomized and counterbalanced. Once all the cups were turned over, the experimenter removed her hands behind the curtain and the owner released the dog. The dog was then allowed to knock over two cups. As soon as the dog knocked over two cups, the experimenter put her hand on the third cup so that the dog could not knock over this cup, and the dog was called back by the owner. The inter-trial interval was 10 s.

#### Variables

The main inhibition measure for this test was the ratio between correct choices (i.e., knocking over both baited cups within a trial; e.g., Amici et al., 2008) in the control (adjacent cups baited) and experimental condition (non-adjacent cups baited). In addition, we calculated the number of correct choices in both conditions separately and coded the latency to make choices (i.e., time from release until second cup was chosen) separately for both conditions. Two dogs were afraid of knocking over the cups and did not complete this test.

### Buzzer test

**Figure 3:**
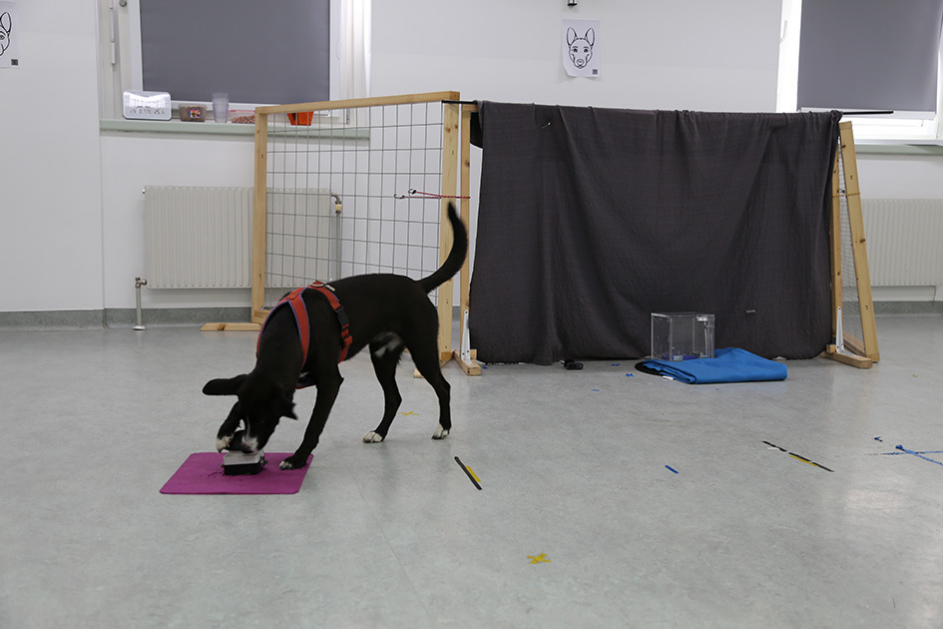
**Setup for test trials in the buzzer test**. The dog presses the buzzer and the box containing the reward is opened.

In this test, the dogs were trained to press a buzzer with either the nose or paw in order to open an opaque box that contained a food reward. In the actual test, the buzzer was positioned away from the baited and transparent box, consequently the dogs needed to turn away from the visible but inaccessible reward to gain access to it by pressing the buzzer (Figure 3). Hence, dogs needed to inhibit the urge to approach and manipulate the box to get the reward directly but instead move away from the reward in order to gain access to it. This test was designed to measure the dogs' motor inhibition abilities but also involved aspects of cognitive inhibition.

#### Setup

A transparent Plexiglas-box (25 × 25 × 25 cm) with a latch that could be opened via a string attached to the opposite side of the box was used for this test. The box was made opaque for the training by attaching pieces of paper to the inside walls, which could easily be removed again for the test. Next to the box we positioned a buzzer (Eaton® FAK-S/KC11/I) attached to a pink fabric pad to stabilize it (see Figure 3). The box was positioned in front of a curtain on a blanket (to reduce the sound of the latch opening, which could scare the dogs). Behind the curtain, which was hung over a wooden frame (1.5 × 2 m), a second experimenter was hiding, who opened the box via an attached string. In order to avoid that dogs interacted with the second experimenter, we placed two fences on either sides of the curtain. The owner sat on a chair 2 m away from the box.

#### Training

In the first training step, dogs were trained to press the buzzer. Positive reinforcement (i.e., clicker and dry food) was used to train the dogs to place their paw on top of the buzzer, which was positioned on the floor. This step was repeated until the dog reliably pressed the buzzer each time the experimenter pointed toward it, in at least five consecutive trials. In the next training step, the dogs were familiarized with the box and its opening movements and sounds. In doing so, the dogs could first retrieve some treats from inside the box, and then the experimenter opened the latch on the front and rewarded the dog each time the latch moved. This training step was repeated until the dogs showed no signs of anxiety when the box opened. In the final training step, the dogs learned to associate pressing the buzzer with the opening of the box. The buzzer was positioned 50 cm next to the opaque box on either the left or the right hand side (the sides were counterbalanced between dogs and the buzzer remained in the same position throughout the test). The owner was instructed to restrain their dog while the experimenter established eye contact with the dog, and said “look,” whilst placing a piece of sausage (2 cm diameter) inside of the opaque box on a blue plastic lid (15 cm diameter), and then closed the box. After the experimenter took a step back to within 1 m of the box and looked toward the box, the owner released the dog and remained silent and motionless until the dog retrieved the reward. As soon as the dog pressed the buzzer, its behavior was marked using a clicker, and the box was opened from behind the curtain by the second experimenter, so that the dog could retrieve the piece of sausage from inside the box, and the next trial begun. If the dog did not press the buzzer within 30 s, the experimenter helped the dog by pointing toward the buzzer. After each successful trial (i.e., without the experimenter helping the dog), the experimenter moved one additional step further away from the buzzer until she was 3 m away. When a dog pressed the buzzer without the experimenter's help in seven consecutive trials, the training criterion was accomplished and the test trials started (11.0 ± 0.6 trials were needed to reach training criterion). A maximum of 20 trials were conducted on one test day and if a dog did not pass criterion, the test was repeated on another day. Two training sessions were conducted (one session per test day), and if the dog did not pass criterion in the second session, the test was terminated and the next test started on the subsequent test day (*N* = 10 dogs).

#### Test procedure

In the *test trials*, the buzzer was positioned 2 m away from the box and the dogs' start position. Additionally, the box was made transparent by removing the attached paper sheets. The owner held the dog by its collar while the dog could watch the experimenter baiting the apparatus. During the first trial, the experimenter touched the buzzer once before baiting the box. This procedure of touching the buzzer was only conducted in the first trial, or as long as the dog was not successful (i.e., not pressing the buzzer within 1 min). As during the training, the experimenter then showed the dog the piece of sausage before placing it in the box, closing it and walked around the buzzer to a location behind the owner without making any eye contact with the dog. As soon as the experimenter reached this spot, the owner released the dog and the trial started. When the dog pressed the buzzer, the box opened immediately, allowing the dog to eat the reward and then the next trial started. If the dog did not press the buzzer within 60 s, the dog was not rewarded, the trial was terminated and the next trial started after a 30 s break. A total of five test trials were conducted.

#### Variables

The main measure of inhibitory control in this test was the time dogs spent in proximity to the box (within a 1 m radius). Additionally, we measured the latency to press the buzzer (i.e., time from releasing the dog until it pressed the buzzer; the maximum duration was limited to 60 s), the duration of manipulating the box (i.e., scratching with paw, pushing with nose) and the number of successful trials (buzzer pressed within 60 s). Some dogs (*N* = 12) did not complete this test because they were either afraid of the sound the box made when it opened or they did not learn to press the buzzer during the training phase.

### Delay of gratification test

In this test, we used the same setup and procedure as Brucks et al. (2017). Dogs were given the choice between receiving an immediate low quality reward and a visible, but inaccessible delayed high quality reward. Dogs could only gain access to the high quality reward if they could refrain from eating the low quality reward and instead wait for the better reward, hence measuring the dogs' self-control abilities. The main inhibition measure for this test was the maximum delay that each dog tolerated.

#### Setup

We used the exact same setup as Brucks et al. (2017) and included the performance from 15 dogs from the previous study without retesting them again, and the 22 additional dogs, which were recruited for the current study (30 dogs did not participate in the DG task). Dogs were placed in an enclosure (ca. 2 m^2^) built out of three wooden frames with heavy weight welded mesh. The front fence had one hole cut out on the bottom to allow the movement of two bowls in and out of the enclosure. A curtain attached to a wooden frame was positioned in front of the enclosure (i.e., 40 cm distance from the front fence), to hide the experimenter and owner during testing. Rewards were delivered via two bowls (one white and round bowl (15 cm diameter), and one black and rectangular bowl (15 × 7 cm), attached to 1 m long sticks, which allowed the experimenter to manipulate them from behind the curtain.

#### Procedure

Prior to the test, we conducted food preference tests with each dog in order to establish a high quality reward (HVR) and a low quality reward (LVR; see Supplementary Material for details on procedure). The different types of rewards were cut into pieces with a similar size of approximately 1.5 × 1.5 cm. In the next step, the dogs were familiarized with two bowls, which differed in their color (black and white) by repeatedly presenting them to the dogs. While the owner held the dog on a leash, both baited bowls were moved toward the dog (i.e., within 30 cm radius), allowing the dog to sniff them. The bowls were then moved sideways (i.e., 80 cm equidistant to dog) before the dog was released and allowed to make a choice. Twelve trials were conducted and the sides of the rewards were alternated between trials. These trials were solely conducted to familiarize the dog with the bowls and the fact that the rewards are presented on them, hence no learning criterion was set.

Following this procedure, the *training trials* commenced. The owner and dog entered the test enclosure. Two types of trials were conducted during the training session: demonstration trials (i.e., the owner restrained the dog from eating the immediately available LVR by silently holding the dog's collar until the HVR bowl entered the enclosure; five trials) and test trials (i.e., the dog was free to choose and the owner remained passive; five trials). If a dog waited for the HVR in at least three test trials, it passed on to the test session, however, if the dog did not reach this criterion, another training session was conducted. The inter-trial interval was about 8 s. In total, no more than four training sessions were run and if a dog did not reach criterion, the delay of gratification test was terminated and it was noted that the dog reached the maximum delay stage of 0 s (*N* = 15 dogs).

In the *test sessions* the delay between the presentation of the LVR and the HVR reward was slowly increased starting with 2, then 5, 10, and up to 20 s. In order to familiarize the dogs with the upcoming delay duration, each test session started with four demonstration trials, in which the owner restrained the dog from eating the LVR. After the demonstration trials were completed, the owner left the enclosure and hid together with the experimenter behind a curtain throughout the test session. Ten test trials were conducted per session and it was noted whether the dog waited for the HVR or consumed the LVR immediately. The inter-trial interval was 8 s, similar to Leonardi et al. (2011). If a dog waited for the HVR in at least three trials, the dog passed on to the next delay stage. If a dog did not reach this criterion, the test session was repeated after a short break. A maximum of 4 test sessions were conducted per delay stage and if a dog did not reach criterion within those sessions, the test was terminated. No more than 3 test sessions were conducted per test day. If dogs successfully waited in the 20 s delay test, the test was likewise terminated. This maximum delay stage was chosen because we observed in our previous study (Brucks et al., 2017), that this delay seemed to be a specific turning point for dogs: either dogs failed before or at 20 s (63% of dogs) or they succeeded and reached much higher delay stages of at least up to 110 s (mean: 250 s). Since dogs in the current study were also tested in the other inhibition tests, we needed to keep the duration of the tests within a feasible time frame and thus limited the maximum delay stage to 20 s.

### Reversal learning test

**Figure 4:**
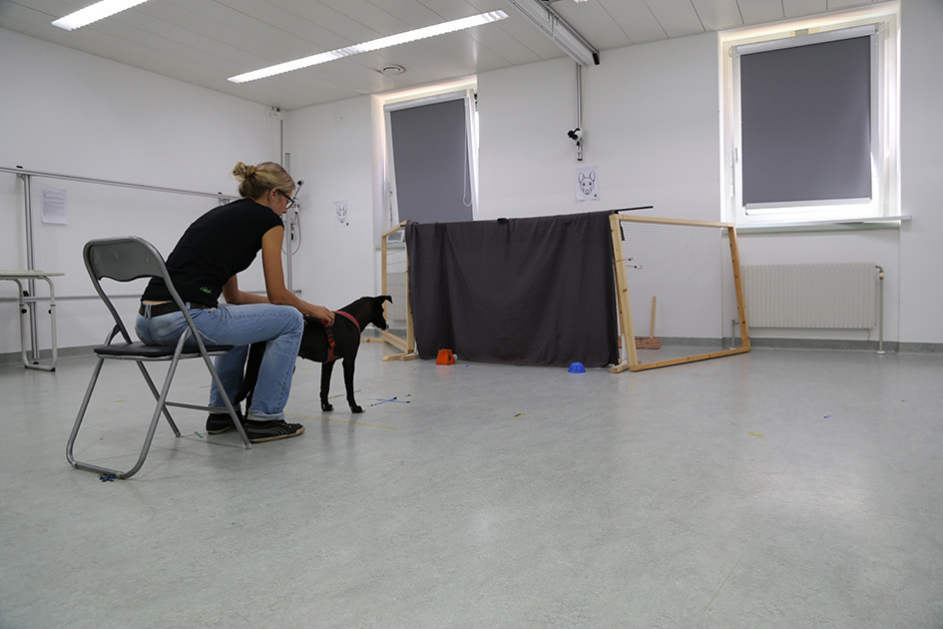
**Setup for reversal learning test**. Rewards were hidden under either the orange or the blue object depending on the test group. The owner sat and held the dog 2 m behind the objects.

In this test, dogs were first taught to discriminate between two different objects (S+ and S−) and to associate one object with a reward (S+; see Figure 4). In a second step, the reward contingencies were reversed, such that the previously positive object (S+) was no longer paired with a reward, while choosing the negative object (S−) was now rewarded. Consequently, dogs needed to inhibit their learnt behavior of approaching the previously rewarded object (S+) and instead approach the other one (S−).

#### Setup

Two objects (an orange, rectangular, felt object and a blue, round, plastic object) were used for this test. The experimenter hid behind a curtain (to reduce the potential influence of social cues on dogs' performance), and observed the dogs via a webcam connected to a laptop behind the curtain. In order to prevent dogs from interacting with the experimenter, two fences were built around the experimenter. A webcam was mounted to the wooden frame and positioned such that the objects were in view. The owner was seated on a chair 2 m away from the curtain and instructed to release the dog from this position without any gestural or verbal comments (see Figure 4). Since both objects were baited through the course of the test (and often within a test day), and hence should have had equal amounts of scent on them, we did not actively control for scent cuing.

#### Warm-up trials

Each phase started with four warm-up trials, in which the dog was presented only with the positive/baited object (S+). Half of the dogs experienced the blue object as the positive object and the other half of dogs had the orange as positive. In these trials, the experimenter presented a piece of sausage to the dog by putting her arms under the curtain and waving the treat in front of the curtain. Then she placed the overturned positive object on top of the treat and removed her hands behind the curtain. At this point, the owner released the dog. In order to facilitate the learning performance of dogs, a secondary reinforcer (clicker) was used as soon as the dog touched the object. After the click, the experimenter lifted the object and the dog was allowed to eat the reward, the owner called the dog back and the next trial started. The majority of dogs (96%) were familiar with clicker training, for the non-clicker trained dogs, verbal praise substituted the clicker during this test.

#### Test procedure

The experimenter hidden behind the curtain (see Figure 4) baited the positive object (S+) then simultaneously pushed both objects out from the front of the curtain. As soon as the experimenter withdrew her hands behind the curtain, the dog was released. If the dog chose the positive object (S+) by touching it with its nose, the experimenter pressed the clicker, a click sound was emitted, and the experimenter lifted the object. However, if the dog chose the negative object (S−), no click was emitted and the experimenter lifted the object, allowing the dog to experience that no reward was hidden under it. In addition, the positive object (S+) was quickly lifted, allowing the dog to see where the reward was hidden but without giving it the chance to obtain the reward. After the dog ate the reward after a successful trial or witnessed where it was hidden (when they chose the incorrect object), the dog was called back and the next trial started. Twelve trials were conducted per session with an inter-trial interval of 10 s. A dog reached criterion in this *acquisition phase* if it chose S+ in at least 9 trials (binomial: *p* = 0.02) within one session. A maximum of 4 sessions were conducted with no more than 2 sessions per test day and if a dog did not reach criterion within 5 sessions, it was excluded from this test (*N* = 3 dogs).

Only dogs that reached criterion in the acquisition phase continued with the *reversal phase* (*N* = 64 dogs; 3 dogs did not reach criterion). The reversal phase started after a short break following the last association trial, with four warm-up trials following the same procedures as before. However, in this case, no clicker was used and the previously negative object (S−) was now baited. Following the warm-up trials, the reversal phase began. As before, the experimenter baited the objects behind the curtain and pushed both forward at the same time. The dog was released when the experimenter removed her hands, and a choice was noted when a dog touched an object with its nose. The dog was allowed to eat the reward if the choice was correct (i.e., approaching S−). If the choice was incorrect (i.e., approaching S+), the chosen object was lifted and the baited object was quickly lifted afterwards in order to show the dog where the reward was hidden. Performance was evaluated in one 12-trial session without setting a learning criterion.

#### Variables

The main inhibition measure for this test was the ratio between the number of correct choices (i.e., choosing the positive object), in the last acquisition and in the reversal phase. In addition, we calculated the number of correct choices separately (last acquisition and reversal phase), and coded the latency to make choices (i.e., time from release to touching an object), as well as the duration dogs spent in the choice area (within 1 m to objects) before they made a choice.

### Analyses

All tests were video recorded and then coded using the program Solomon Coder beta (© 2015 by András Péter; http://solomoncoder.com/). Data was analyzed using R (R Core Team, 2014, Version 3.1.2), and SPSS Statistics 22 (IBM Corporation). The dogs' performance in each test was first analyzed separately using Wilcoxon tests as well as linear models in R. In order to account for the differences in variables across tests, the inhibition variables from the single tests as well as the variables from the impulsivity questionnaire were *z*-transformed (thus allowing a comparison on the same scale), before correlating the standardized measures with each other using Spearman correlations.

To further investigate the potential underlying processes involved, a Principle Component Analysis (PCA) (using SPSS) was carried out for each test, entering the raw measures of the behavioral variables coded. This allowed us to meaningfully reduce the number of variables for each test. In a second step, a higher-order PCA was performed on all the components extracted from the separate tests, to investigate the relationship between the components derived from the single tests. We utilized the method described by Turcsán et al. (submitted) to calculate component scores by making a template on a reduced sample, which did not allow any missing values at the variable level. All PCAs were run using Quartimax rotation and components with an Eigenvalue of at least 1 were retained in the final solution. From the results of the individual test PCAs and the higher-order PCA, which showed the internal component structure, we calculated component scores for each individual dog in order to allow for missing values, using the following steps: First, z-transformations were conducted on the raw variables. Second, the subtest-level component scores were derived by calculating the mean of the variable loadings as found in the initial template PCA. Only loadings of at least 0.5 were considered and variables with negative loadings were included in the calculation by multiplying by −1. If more than one-third of the variables, which loaded on the same component, were missing for a dog, this component score was not calculated for that individual. In the final step, the higher-order components (i.e., over all the different inhibition tests) were derived as before by calculating the mean of the single test-level components. Only components with a loading of at least 0.5 were considered and no more than one-third of the variables loading on one component were allowed to be missing. Finally, we ran linear models (LM) on the final components to look at possible influencing factors (e.g., age, sex) and the link between the components and the scores of the questionnaire. LMs were run in R using a stepwise model reduction method to retain only significant effects in the model.

Reliability coding was conducted on 20% of the videos, revealing good consistency between coders [Intra-class correlation coefficients (ICC) for continuous variables: all ICC (consistency) > 0.7; Cohen's kappa for frequencies: all kappa > 0.9].

## Results

### Correlation between inhibition measures across tests

Not all dogs succeeded in each task due to fear of the apparatuses (middle cup: *N* = 2, buzzer: *N* = 6) or an inability to learn the task contingencies (buzzer: *N* = 4, reversal-learning: *N* = 3). Moreover, not all dogs were tested in the delay of gratification task due to time restraints (*N* = 30). Consequently, data is available for 67 dogs in the box test, 65 dogs in the middle cup test, 57 dogs in the buzzer test, 37 dogs in the delay of gratification test, and 64 dogs in the reversal-learning test. Nonetheless, when considering the main measure of inhibition for each test, no correlation in the inhibitory control performance of dogs across tests emerged (see Table 1). The impulsivity score obtained from the questionnaire also did not correlate with test measures of inhibitory control, with one exception—the delay tolerated in the delay of gratification test was negatively correlated with the overall questionnaire score (*r*_*s*_ = −0.39, *p* = 0.02) indicating that dogs with assumed lower impulsivity (or higher inhibitory control abilities), have more difficulties in tolerating high delays.

**Table 1:** **Spearman correlation matrix between z-transformed inhibition measures from single tests and the measures obtained from the questionnaire (***N*** = 57 dogs)**.

**Inhibition measures**						**DIAS questionnaire**			
	**Box**	**Middle cup**	**Buzzer**	**Delay Gratification**	**Reversal learning**	**Overall**	**Factor 1**	**Factor 2**	**Factor3**
	**Errors**	**Ratio control/exp**.	**Vicinity to box**	**Max. delay**	**Ratio acquisition/reversal**	**Impulsivity score**	**Behav. regulation**	**Reaction to novelty**	**Responsiveness**
B		*r_*s*_* = 0.02	*r_*s*_* = 0.14	*r_*s*_* = 0.15	*r_*s*_* = 0.12	*r_*s*_* = 0.15	*r_*s*_* = 0.15	*r_*s*_* = 0.02	*r_*s*_* = 0.15
MC			*r_*s*_* = − 0.12	*r_*s*_* = 0.07	*r_*s*_* = 0.03	*r_*s*_* = −0.01	*r_*s*_* = 0.02	*r_*s*_* = 0.08	*r_*s*_* = 0.05
BUZ				*r_*s*_* = 0.28	*r_*s*_* = −0.20	*r_*s*_* = −0.10	r_*s*_ = 0.02	*r_*s*_* = 0.06	*r_*s*_* = −0.08
DG					*r_*s*_* = 0.04	*r_*s*_* = −0.39^*^	*r_*s*_* = −0.33	*r_*s*_* = 0.14	*r_*s*_* = −0.33
RL						*r_*s*_* = − 0.02	*r_*s*_* = −0.02	*r_*s*_* = 0.05	*r_*s*_* = 0.11

* *p < 0.05; B, Box, BUZ, Buzzer, DG, Delay of Gratification, MC, Middle Cup, RL, Reversal Learning*.

#### Box test

Dogs spent more time close to the box when it was transparent (median = 29 s, IQR = 37.75) compared to the training trials with an opaque box (median = 14.7 s, IQR = 8.2; Wilcoxon Test: *z* = 4.77, *N* = 67, *p* < 0.001). Moreover, dogs tended to commit more errors (i.e., touching the box's surface with either paw or nose) in the test trials when the box was transparent (median = 3, IQR = 10.5) compared to the training trials when the box was covered (median = 3, IQR = 5; Wilcoxon Test: *z* = 1.66, *N* = 67, *p* = 0.078). Individual performances differed substantially between 0 and 75 errors committed during the test and 7.8–199.4 s spent close to the box. Additionally, the reward location affected the latency to retrieve the reward out of the box. Dogs needed longer to find the open side when the reward was placed deep inside the box (median = 24.8 s, IQR = 37.55) compared to a central position (median = 13.8 s, IQR = 11.55; Wilcoxon Test: *z* = 4.95, *N* = 67, *p* < 0.001). All the above analyses suggest that the transparency of the box and the location of the food placed additional requirements on dogs' capacity to solve the task.

The PCA revealed that the two variables measuring the latency to retrieve the reward in the deep and center conditions grouped on one factor, whereas the number of paw and nose touches grouped on another factor (Table 2). We labeled the first factor “inflexibility” since the latency to find the opening of the box depended on the dogs' explorative abilities, but also on their flexibility to try out more options to get access to the box. The second factor was labeled “perseveration” as this factor included the variables measuring the number of errors (surface touches) dogs made during the test. These two factors explained 74.2% of the observed variation in this test.

**Table 2:** **Factors derived from PCA on the variables measured in the box test**.

	**Components**	
**Variable**	**Inflexibility**	**Perseveration**
Latency deep condition	0.87	
Number of successful trials	−0.87	
Duration close to box	0.86	
Latency center condition	0.85	
Paw errors		0.85
Nose errors		0.84
% variance	50.18	23.89
Cronbach's α	0.88	0.61

*KMO = 0.53; Bartlett: χ^2^ = 178.4, df = 15, p < 0.001*.

#### Middle cup test

Dogs were more successful (= knocking over both baited cups) in the control trials (median = 6 correct choices, IQR = 2) compared to the test trials (median = 1 correct choice, IQR = 4; Wilcoxon Test: *z* = 5.96, *N* = 65, *p* < 0.001). Large individual differences in performance were revealed with 3–10 correct choices in the control trials and 0–10 correct choices in the experimental trials. Moreover, dogs took more time to make their choices in the test (median = 66 s, IQR = 46.6, range: 32.2–206.2 s) compared to the control condition (median = 60 s, IQR = 44.8, range: 30–147 s; Wilcoxon Test: *z* = −3.42, *N* = 65, *p* < 0.001), suggesting that, as predicted, task difficulty increased in test trials.

The PCA showed that the latency to make a choice in the control as well as in the experimental condition grouped together on one factor, which we labeled “decision time.” Whereas the number of correct choices in both conditions grouped on another factor, which received the label “attention” as dogs needed to pay attention to the location of the hidden rewards before making their choices in order to be successful in both conditions (see Table 3). Those two factors cumulatively explained 83.2% of the observed variation.

**Table 3:** **Factors derived from PCA on the variables measured in the middle cup test**.

	**Components**	
**Variable**	**Decision time**	**Attention**
Duration close to cups	0.98	
Latency to choice exp. cond.	0.94	
Latency to choice control cond.	0.91	
Correct choices control trials		0.89
Correct choices exp. trials		0.74
% variance	59.84	23.34
Cronbach's α	0.94	0.58

*KMO = 0.63; Bartlett: χ^2^ = 09.44, df = 0, p < 0.001*.

#### Buzzer test

Dogs learned the association between buzzer pressing and box opening within a median of 10 trials (IQR = 5). They manipulated the box for a longer time if it was transparent (test trials; median = 3.64 s per trial, IQR = 6.25) compared to when it was still covered (training trials; median = 0.17 s per trial, IQR = 0.50; Wilcoxon Test: *z* = 6.42, *N* = 57, *p* < 0.001). Additionally, the latency to press the buzzer was significantly longer in test trials (median = 4 s per trial, IQR = 4.76) than in training trials (counting only trials in which the dogs required no help from the experimenter: median = 0.75 s per trial, IQR = 0.61; Wilcoxon Test: *z* = 6.42, *N* = 57, *p* < 0.001). These results show that dogs were more inclined to stay close to the box during the test trials and hence validates that the transparent condition was indeed more difficult for the dogs. Individual performances in the buzzer test were quite diverse, ranging from 0 to 123.8 s of box manipulation per trial and a range of 3.36–51.40 s latency to success per trial. Dogs showed no evidence of learning throughout the test trials, as they did not become faster at pressing the buzzer across trials [LM: *F*_(1, 278)_ = 2.36, *p* = 0.13].

A PCA on the measured variables for this test revealed that they grouped on one factor explaining a variance of 70.2% (see Table 4). We labeled this factor “persistence,” since the variables that strongly loaded on it all measured behaviors associated with staying close to the box.

**Table 4:** **Factor derived from PCA on the variables measured in the buzzer test**.

**Variable**	**Component**
Duration close to box	0.91
Latency to success	0.91
Number of successful trials	−0.83
Duration manipulate box	0.69
% variance	70.2
Cronbach's α	0.85

*KMO = 0.61; Bartlett: χ^2^ = 183.62, df. p < 0.001*.

#### Delay of gratification test

Of the 37 dogs, which were tested in the delay of gratification test, 19 dogs (51.4%) did not wait for the lowest delay stage (i.e., 2 s), whereas 10 dogs (27%) reached the maximum delay stage of 20 s. Two dogs reached the 2 s stage, four dogs the 5 s stage and three dogs tolerated a maximum of 10 s. On average, the dogs waited successfully in 10 trials (IQR = 20.75, range 0–37 trials). No data reduction method was applied to this test since only two variables were measured (the number of successful trials and the maximum delay stage reached).

#### Reversal learning test

Dogs took more time to make a choice in the reversal phase (median = 11.60 s, IQR = 3.18, range: 15.2–81.6 s) compared to the acquisition phase in which they reached criterion (median = 10.50 s, IQR = 2.2, range: 15.6–127.6 s; Wilcoxon Test: *z* = −4.69, *N* = 64, *p* < 0.001). They were significantly less successful in the reversal phase (median = 3 correct out of 12 trials, IQR = 3; 28.9% correct; range: 0–9 correct choices) compared to the acquisition phase, when considering only the session in which they reached criterion (median = 10.5 correct out of 12 trials, IQR = 1; 86.3% correct choices; Wilcoxon Test: *z* = 6.64, *N* = 64, *p* < 0.001). Dogs needed on average 2.10 ± 0.14 sessions to reach the learning criterion, and the dogs' capacity to learn the association, as measured in the number of sessions needed to reach criterion, was not correlated with their performance in the reversal phase (Spearman correlation: *r*_*s*_ = 0.11, *N* = 64, *p* = 0.40).

The variables measuring the latency to make a choice in the acquisition and in the reversal phase (as well as the duration dogs spent in the choice area), grouped together on one factor (labeled “choice time”). In contrast, the number of correct choices in the last acquisition phase and reversal phase grouped together on another factor (one with a positive and the other with a negative loading). This factor was labeled “certainty,” since given the opposite loadings, it appears to measure the dogs' tendency to stick with their initial preference even after repeated reinforcement for the other option (see Table 5). These factors explained 62.1% of the variation in this test.

**Table 5:** **Factors derived from PCA on the variables measured in the reversal-learning test**.

	**Components**	
**Variable**	**Choice time**	**Certainty**
Latency to choice in reversal phase	0.86	
Latency to choice in acquisition phase	0.77	
Duration close to objects	0.63	
Correct choices in last acquisition		0.87
Correct choices in reversal phase		−0.70
% variance	39.24	22.84
Cronbach's α	0.61	0.33

*KMO = 0.61; Bartlett: χ^2^ = 37.47, df = 10, p < 0.001*.

### Overall PCA analyses

We conducted a PCA on the factors derived from the individual inhibition tests. Since we considered only one variable from the delay of gratification test, we did not perform a PCA on this test, but rather entered the standardized *z*-value into the overall PCA.

The overall PCA revealed three underlying components explaining a cumulative variation of 63.5% (see Table 6). The first component included the maximum delay reached in the delay of gratification test, the “perseveration” factor and the “inflexibility” factor from the box test, and the “persistence” factor from the buzzer test. Considering that those factors all measure the dogs' persistency in getting access to rewards, we labeled this component “persistency.” The second component included the “certainty” factor from the reversal-learning test as well as the “attention” factor from the middle cup test. Since both factors involved measures of dogs' flexibility or rather compulsivity in maintaining their inappropriate choice behavior (i.e., reversal learning test: sticking with their preferred object; middle cup test: choosing adjacent cups), we labeled this component “compulsivity.” The third component included the “inflexibility” factor from the box test and the “decision speed” factors from the middle cup and reversal-learning test. Since those factors describe the dogs' decisiveness, we labeled this component “decision time.”

**Table 6:** **Components derived from PCA on the factors measured in the five inhibition tests**.

	**Components**		
**Variable**	**Persistency**	**Compulsivity**	**Decision time**
DG: maximum delay	0.77		
C: perseveration-errors	0.77		
BUZ: persistence-vicinity	0.62		
B: inflexibility	0.53		0.52
RL: certainty		0.86	
MC: attention		0.80	
MC: decision speed			0.81
RL: decision speed			0.55
% variance	26.49	20.30	16.85
Cronbach's α	0.64	0.35	0.43

*KMO = 0.51; Bartlett: χ^2^ = 46.47, df = 28, p = 0.02. DG, Delay of gratification, B, Box test, BUZ, Buzzer test, RL, reversal learning test, MC, middle cup test*.

### Validation with questionnaire and influence of age and sex

In order to determine whether the derived components can be explained by the measures obtained from the self-control questionnaire (DIAS) or by other individual characteristics (in particular the age or sex of the dogs), we ran separate linear models for each component (see Table 7). Whereas, the first component “persistency” was not linked to any of the other measures, our second component “compulsivity” was negatively affected by age (LM: β = −0.07, *SE* = 0.03, *t* = −2.46, *p* = 0.02), and the third component “decision time” was negatively affected by the third factor of the questionnaire labeled “responsiveness” (LM: β = −1.53, *SE* = 0.63, *t* = −2.42, *p* = 0.02).

**Table 7:** **Summary of linear model outputs for relationship between components and age, sex and scores derived from DIAS questionnaire**.

			**Impulsivity Questionnaire**			
	**Age**	**Sex**	**Overall score**	**Behavioral regulation**	**Reaction to novelty**	**Responsiveness**
Persistency	*F* = 0.37, *p* = 0.55	*F* = 1.17, *p* = 0.28	*F* = 2.95, *p* = 0.09	*F* = 0.38, *p* = 0.54	*F* = 3.35, *p* = 0.07	*F* = 1.35, *p* = 0.25
Compulsivity	*F* = 6.07, *p* = 0.02	*F* = .081, *p* = 0.37	*F* = 1.15, *p* = 0.29	*F* = 0.42, *p* = 0.52	*F* = 0.85, *p* = 0.36	*F* = 0.36, *p* = 0.55
Decision time	*F* = 0.49, *p* = 0.49	*F* = 1.72, *p* = 0.20	*F* = 0.25, *p* = 0.62	*F* = 1.25, *p* = 0.27	*F* = 0.10, *p* = 0.75	*F* = 5.84, *p* = 0.02

*Significant results are highlighted in bold letters*.

## Discussion

Dogs' inhibitory control abilities were assessed in a battery consisting of tests frequently used in the literature. We found that in line with results from previous studies, the tests captured dogs' inhibitory control abilities. More specifically, in each test condition (i.e., involving an increased salience of rewards) it was more effortful for the dogs to solve the tasks (in terms of errors and time) compared to the basic training/control conditions. Nonetheless, the supposed “inhibition” measures obtained from the five single tests did not correlate with each other, indicating that those five inhibitory control tasks do not measure the same behavior. Nor did they show any meaningful relationship with the scores from a previously validated dog impulsivity questionnaire. Consequently, in order to understand whether any underlying structure exists in dogs' performance across tasks, we conducted a PCA on the various measures in each task. We found three components—persistency, compulsivity and decision time, which best captured the underlying structure of the test battery. These three components explained the variation within dogs' behavior across tasks, but no single element emerged as a measure of inhibitory control *per se*. In addition, individual characteristics as well as the owner-reported questionnaire only modestly captured the variance in our three-component solution.

When looking at each test separately, dogs' performance clearly differed between the training/control condition and the actual inhibitory control eliciting test condition. In the box test, dogs needed longer to find the open side and committed more perseverative errors, if the box was transparent, indicating that the visibility of the reward strongly affected dogs' ability to inhibit reaching for the reward directly. This indicates that prepotent responses (i.e., touching the surface repeatedly) increased when the salience of the reward increased (i.e., visibility in transparent test trials) even though dogs experienced tactile feedback from the solid surface. This is in line with results from monkeys and young children (e.g., Diamond, 1990; Hauser, 1999), who similarly committed more perseverative behaviors when the reward was visible through a transparent barrier. The number of perseverative errors committed by the dogs (mean: 10.2 errors) was similar to rates found in 10-month-old children (11.4 errors; Lockman and Adams, 2001) and squirrel monkeys (11.7 errors; Parker et al., 2005). Likewise, dogs' behavior in the middle cup test was consistent with the results from other studies (e.g., Amici et al., 2008; Müller et al., 2016) and dogs had more difficulties to inhibit knocking over the empty cup if rewards were hidden in non-adjacent locations. Dogs' performance in the buzzer test cannot be compared to other species since it has been newly developed. Furthermore, the delay of gratification task also revealed that most of the dogs had difficulties in inhibiting their behavior to eat the immediately offered reward option, in favor of waiting for the better reward and only half of the dogs succeeded in the minimum delay stage of 2 s, whereas 27% of the dogs reached the maximum delay of 20 s. Consequently, the dogs showed lower delay of gratification abilities than many other species tested in this paradigm (e.g., chimpanzees: Dufour et al., 2007; corvids: Hillemann et al., 2014; macaques: Evans and Beran, 2007). Finally, in the reversal-learning task, dogs' performance declined when the objects were reversed, indicating that this task was indeed measuring the dogs' abilities to inhibit choosing the previously rewarded object. Importantly, the results from the reversal learning test need to be interpreted with caution for two reasons: firstly, since we removed the clicker component from the reversal phase, dogs' performance in this phase could have been impaired, and secondly, our study contained procedural differences to the commonly used experimental design. For example, there are no warm-up trials and the reversal phase often involves more than one reversal session (e.g., Tapp et al., 2003; Bond et al., 2007; Beran et al., 2008).

Taken together, the results from each task reveal that dogs do possess some inhibitory control abilities, as the majority of dogs could succeed in each task. While each inhibition task captured the dogs' behavior in line with previous research, it is also clear, and not surprising, that the “inhibition” behaviors measured in each task were not correlated with each other, indicating that each test measures different inhibitory control abilities. In other words, while we found-enormous individual variation within each task, dogs that were particularly good in one task, did not do well in another. This is in line with previous studies both with humans (e.g., Tsukayama et al., 2012) and with dogs (Bray et al., 2013; Marshall-Pescini et al., 2015; Müller et al., 2016), and suggests that dogs' inhibitory control abilities are highly context specific. Moreover, aspects of motivation, learning, and experience are employed in the different inhibition tasks to differing extents. For example, increased motivation is likely to arise when the food is visible (i.e., in the box, buzzer, middle cup, and delay of gratification tests), which can potentially negatively influence the dogs' inhibition abilities in those tests where inhibiting going toward the food is necessary. However, in the delay of gratification task, increased saliency of the higher reward, combined with a heightened motivation, could result in a superior performance of the animal, due to the ability of the dog to focus on the target stimuli. In contrast, the motivational aspect of visible food is not present in the reversal-learning test. Furthermore, differences in learning abilities might explain why performance in tasks measuring a more “spontaneous” response (i.e., box and middle cup test) is not correlated with tasks that involve a learning component (i.e., buzzer and reversal learning test). In connection to the learning aspect, individual differences in experience with certain features of a task may occur, and these are difficult to control for, e.g., some dogs might have more experience with transparent surfaces than other dogs due to everyday life situations in the household or on walks. Considering that motivation, learning, and experience are potentially involved to a variable degree in the different inhibition paradigms, it remains of major importance to extract the actual inhibition component from the tasks.

In humans, inhibitory control is explained as a collection of behavioral processes instead of a single, distinct ability, and based on current results the same seems to be true for dogs. In order to understand whether the dogs' behavior across tests could be categorized, revealing an underlying structure of inhibition measures, we conducted principle component analyses. Interestingly, the underlying components explaining the dogs' performance in the inhibition tests (persistency, compulsivity, decision time), were similar to those described in the human literature (e.g., Miller et al., 2004; see Evenden, 1999 for a review).

*Persistency*, for example, seems to be linked to inhibitory control in a way that more persistent individuals have more problems in exhibiting inhibitory control (e.g., Buss and Plomin, 1975; Diamond and Gilbert, 1989). Also, in our study, measures of persistence from three tests (box test: number of surface touches with either paw or nose and time spent close to box; buzzer test: time spent in vicinity of box; delay of gratification test: maximum delay tolerated), loaded on one component, which negatively affected performance. While measures of motor inhibition have often been shown to be consistent across tasks, at least in humans, the performance on delay of gratification tasks is normally considered as a separate category measuring self-control rather than response inhibition (e.g., Duckworth and Kern, 2011; Beran, 2015). Interestingly, measures of response inhibition and self-control grouped together on one component in our study, and the dogs' performance in the delay of gratification task was negatively related to performance in the two motor inhibition tasks. In delay of gratification tests, individuals waiting for the better reward—hence inhibiting eating the immediately offered reward—are considered to have better self-control/inhibitory control abilities (e.g., Duckworth et al., 2013). However, results from the current study relating measures of inhibition across tasks show the opposite effect, in that dogs that tolerated higher delays committed *more* errors in the box test, and had *more* problems in stepping away from the box to press the buzzer (in the buzzer test). This would seem to suggest that in dogs, the delay of gratification test might be a measure of how persistent they are in focusing on the higher value/delayed reward, rather than refraining from taking the lower value one, potentially capturing a measure of how “motivating” the reward is for the animal. An explanation for this interesting relationship might be that dogs, which are generally highly food motivated, have a higher motivation to gain the delayed but better reward in the delay of gratification test than less food motivated dogs, which might just take whatever reward comes first. Additionally, dogs with a higher food motivation level made more errors in the physically more active box and buzzer tests, because their motivation to engage in physical activity (i.e., manipulating the boxes), and motivation to obtain a reward was higher. However, not all dogs were tested in the delay of gratification task, hence lowering the statistical power of this test. Moreover, we did not find a relationship between the maximum delay and the conventional “inhibition” variables of the box and buzzer test in the correlational approach, consequently the interplay between underlying behaviors that are measured in these tests is not that straight forward. In addition, the delay task was not conducted until the dogs reached their actual point of indifference, but rather only up to a maximum of 20 s. Despite these limitations, the current results provide a starting point for future studies to investigate the interplay between persistency and inhibitory control but also self-control. In addition, future research should investigate whether self-control, as assessed with a delay of gratification task, is substantially different from other inhibitory control tasks (i.e., motor inhibition) in non-human animal species.

Another aspect of inhibitory control, which has been described in humans, is *compulsivity* or rather inflexibility (e.g., Bari and Robbins, 2013), and it is assumed that more flexible individuals have better inhibitory control abilities (e.g., Izquierdo and Jentsch, 2012). Interestingly, our component “compulsivity” included measures from the reversal-learning test as well as the middle cup test. This component composition is quite surprising as the reversal-learning test is considered a test of cognitive inhibition, whereas the middle cup test is considered a test of motor inhibition. Based on the human literature (e.g., Duckworth and Kern, 2011), we would expect these two tests not to group together as they are thought to measure different aspects of inhibitory control. One explanation for this might be that both tests include forced choices, contrary to the other inhibition tests, in which a non-restricted behavior was measured. Consequently, they seem to capture dogs' compulsive behavior in sticking with their choice of the initially rewarded object in the case of the reversal-learning task, and their attention to follow and choose cups that are baited in the middle cup task, instead of always choosing adjacent cups. This indicates that insensitivity to negative feedback plays a role in this component. Additionally, the ability to shift attention to the tasks features necessary for improving performance is also required (i.e., reversal-learning: not paying attention to reversed objects, and middle cup: maintaining attention and switching strategies between control and experimental condition; see also Bari and Robbins, 2013). Interestingly, we found that this compulsivity component was negatively correlated with age. Older dogs showed less compulsive behavior and hence performed better than younger dogs, which is rather surprising as previous studies have found that older dogs are less flexible in their choices (e.g., Tapp et al., 2003; Wallis et al., 2016). This age effect needs a cautious interpretation, since it was mainly driven by the reversal-learning test. And indeed, in an attempt to understand this effect, we found a negative correlation between age and correct choices in the acquisition phase (Spearman: *r*_*s*_ = −0.24, *p* = 0.05), which implies that older dogs did not learn the reward contingencies in the acquisition phase as well as the younger dogs. Possibly, younger dogs learned the reward contingencies more quickly and then had more problems inhibiting their preference in the reversal phase (see also Wallis et al., 2014). Hence, the older dogs' superior performance in the reversal may simply be a by-product of their poorer performance in the association phase: younger dogs struggled more to switch because they had internalized the “rules” more strongly than older dogs. Interestingly, other studies investigating reversal-learning performance do not directly investigate the relationship between acquisition performance and reversal performance, making it difficult to assess whether individuals with a lower acquisition performance are generally better in the reversal phase. According to contemporary instrumental learning theory, two different strategies can be at work when learning new tasks. In the first stage the individual learns the new contingencies in an active and goal-directed way, whereas once the features of the task are learnt, a more automated and habitual control of behavior occurs (see Schwabe and Wolf, 2011 for a review). According to this learning theory, an alternative explanation might be that younger dogs switched to an automated learning strategy during the acquisition phase, since they learned the contingencies more quickly than older dogs. The younger dogs consequently had more problems in changing their learned contingencies in the reversal phase, whereas since older dogs did not learn the initial association as well, they were likely to switch their strategy, which facilitated their performance in the reversal phase allowing them to make more flexible decisions. Alternatively, the removal of the clicker component in the reversal phase could have had a bigger impact on the younger dogs' performance than on the older dogs.

The final aspect emerging from the PCA, which was rather stable across tasks, was the time it took dogs to reach a decision. This was reflected in our final component, which we labeled “*decision time*” and included measures from the box, middle cup and reversal-learning test. In the human literature, decision time has been mentioned as one aspect of inhibitory control, however, more so as a personality dimension involved in inhibitory control (i.e., sensation seeking or urgency; e.g., Buss and Plomin, 1975; Whiteside and Lynam, 2001). The decision time component was negatively correlated with a score for responsiveness from the impulsivity questionnaire. Dogs that were described in the questionnaire as less responsive in terms of environmental awareness (i.e., trainability, reaction time, and interest in novel things), also showed a slower decision time in the test battery. It seems as if this motivational aspect is reliably and consistently assessed with the questionnaire and the inhibition test battery.

To our knowledge this is the first study that carried out an in-depth analysis of inhibition measures in non-human animals, revealing that, at least in dogs, inhibition tasks do not measure a single, unified process but rather different abilities, such as persistency in getting access to rewards, consistency/attention and general speed in making decisions. Considering that other species also exhibited variation in their inhibition abilities across tasks (e.g., wolves: Marshall-Pescini et al., 2015; primates: Amici et al., 2008), it is possible that inhibitory control evolved in a context-specific way rather than as a general ability. Also in humans, inhibitory control abilities have been found to vary across contexts. Accordingly, it has been proposed that humans possess a general ability to cope with impulsivity, but the ability to resist temptation can vary dramatically in different contexts (e.g., Tsukayama et al., 2012). Hence, we suggest that inhibitory control is not a unitary process but is context/domain specific in human and potentially also non-human animals.

Although it remains unclear if the same conclusions hold true for other species, considering these different aspects of inhibitory control, we need to be cautious when drawing conclusions about the involvement of inhibitory control in other cognitive processes. For example, problem-solving (Vlamings et al., 2010), aspects of a species' life (social organization: Reddy et al., 2015; feeding ecology: Vernouillet et al., 2016) or factors affecting its evolution (MacLean et al., 2014). While it remains essential to study the effect of training on inhibitory control abilities, the influence of domestication on inhibitory control also needs to be investigated in future studies. Up until now, no consistencies between dogs' and wolves' inhibitory control abilities have emerged (Marshall-Pescini et al., 2015). Consequently, testing dogs and wolves in a similar setup as in the current study is needed to understand whether the context dependency evolved during domestication, or whether wolves behave similarly.

## Ethics statement

All procedures were approved by the institutional ethics commission of the University of Veterinary Medicine Vienna (Approval number: 10/12/97/2013).

## Author contributions

DB, SM, and FR designed the experiments. DB conducted the experiments and coded videos. DB, LW analyzed the data. DB, SM, LW, LH, and FR wrote the paper.

### Conflict of interest statement

The authors declare that the research was conducted in the absence of any commercial or financial relationships that could be construed as a potential conflict of interest.
