# Supplementary material for: Measures of Dogs' Inhibitory Control Abilities Do Not Correlate across Tasks
Source: Front Psychol. 2017 May 24;8:849. doi: 10.3389/fpsyg.2017.00849 (PMC5443147; doi:10.3389/fpsyg.2017.00849)
Supplement: Supplementary file 1 [file DataSheet1.DOCX]

**Supplementary material**

**Table S1.** Individual characteristics of dogs, missing tests and scores obtained from the impulsivity questionnaire (DIAS)

| # | Name | Age (yrs.) | Sex | Breed | missing | DIAS | Factor1 | Factor2 | Factor3 |
| --- | --- | --- | --- | --- | --- | --- | --- | --- | --- |
| 1 | Achuk | 9.8 | F* | Chesapeake Bay Retriever | - | 0.51 | 0.50 | 0.20 | 0.80 |
| 2 | Aiko | 4.2 | M* | Australian Shepherd | - | 0.62 | 0.50 | 0.32 | 0.76 |
| 3 | Akin | 6.2 | M* | Rhodesian Ridgeback | DG | 0.49 | 0.44 | 0.36 | 0.72 |
| 4 | Akina | 6.7 | F* | Akita Inu | - | 0.57 | 0.54 | 0.56 | 0.45 |
| 5 | Anton | 2.5 | M* | Malinois | DG | 0.44 | 0.34 | 0.20 | 0.72 |
| 6 | Asta | 7.2 | F | Entlebucher Mountain Dog | DG | 0.54 | 0.50 | 0.32 | 0.80 |
| 7 | Baja | 3.7 | F* | Australian Shepherd | - | 0.56 | 0.58 | 0.64 | 0.68 |
| 8 | Barolo | 5.3 | M | Standard Poodle | DG | 0.61 | 0.70 | 0.20 | 0.84 |
| 9 | Bella | 8.2 | F* | Bernese Mountain Dog | - | 0.41 | 0.26 | 0.20 | 0.84 |
| 10 | Benji | 5.3 | M* | Husky – Mix | DG | 0.56 | 0.50 | 0.28 | 0.92 |
| 11 | Brandy | 4.8 | F* | Bernese Mountain Dog | - | 0.64 | 0.58 | 0.40 | 0.80 |
| 12 | Buck | 6.1 | M* | Beagle | - | 0.50 | 0.54 | 0.36 | 0.68 |
| 13 | Cameron | 2.8 | M | Border Collie | - | 0.40 | 0.20 | 0.52 | 0.68 |
| 14 | Charlie | 6.7 | M | Bearded Collie | DG | 0.64 | 0.60 | 0.44 | 0.80 |
| 15 | Chasie | 6.4 | F* | Border Collie | - | 0.42 | 0.30 | 0.20 | 0.96 |
| 16 | Cole | 7.9 | M* | Border Collie | - |  |  |  |  |
| 17 | Cookie^x^ | 3.3 | M | Bearded Collie | BUZ,DG,RL | 0.73 | 0.64 | 0.72 | 0.84 |
| 18 | Daimony | 8.3 | F* | Australian Shepherd | - | 0.50 | 0.46 | 0.36 | 0.64 |
| 19 | Elrond | 6.1 | M* | Chesapeake Bay Retriever | BB | 0.71 | 0.82 | 0.40 | 0.68 |
| 20 | Emely | 3.4 | F* | Bernese Mountain Dog | - | 0.68 | 0.80 | 0.28 | 0.60 |
| 21 | Emily | 7.3 | F* | Border Collie | - | 0.44 | 0.34 | 0.36 | 0.76 |
| 22 | Emma | 2.3 | F | German Shepherd | DG | 0.51 | 0.42 | 0.32 | 0.76 |
| 23 | Esprit | 5.2 | F | Border Collie | - |  |  |  |  |
| 24 | Fibi^x^ | 3.3 | F* | Miniature Pinscher-Mix | BUZ,DG,MC | 0.61 | 0.56 | 0.64 | 0.84 |
| 25 | Flamme | 7.6 | M | Berger des Pyrénées | - | 0.63 | 0.70 | 0.36 | 0.76 |
| 26 | Gatsby | 5.2 | M | Border Collie | - | 0.51 | 0.42 | 0.44 | 0.72 |
| 27 | Gizmo^x^ | 8.9 | M* | Chihuahua - Mix | BUZ,MC | 0.71 | 0.68 | 0.56 | 0.64 |
| 28 | Hanya^x^ | 1.3 | F | German Shepherd | BUZ,DG | 0.59 | 0.60 | 0.28 | 0.64 |
| 29 | Hybie | 6.5 | F* | Labrador - Mix | - | 0.64 | 0.60 | 0.36 | 0.88 |
| 30 | Idefix^x^ | 12.1 | M | West Highland Terrier | BUZ,DG,RL | 0.73 | 0.70 | 0.36 | 0.72 |
| 31 | Iken | 2.3 | M* | Border Collie | DG | 0.43 | 0.38 | 0.44 | 0.80 |
| 32 | Ivory | 7.7 | F | Labrador Retriever | DG | 0.49 | 0.28 | 0.28 | 0.84 |
| 33 | Keksi^x^ | 3.5 | F* | Shepherd - Mix | BUZ,DG | 0.46 | 0.36 | 0.32 | 0.84 |
| 34 | Kilio | 5.5 | M* | Terrier - Mix | - | 0.55 | 0.58 | 0.50 | 0.56 |
| 35 | Kiwi^x^ | 3.6 | F* | Border Collie - Mix | BUZ,DG | 0.49 | 0.44 | 0.44 | 0.84 |
| 36 | Lenny | 6.5 | M | Border Collie | DG | 0.49 | 0.38 | 0.36 | 0.88 |
| 37 | Leopold | 5.8 | M | Petit Brabançon | DG | 0.43 | 0.30 | 0.20 | 0.84 |
| 38 | Lilli | 7.8 | F* | Golden Retriever | DG | 0.60 | 0.62 | 0.60 | 0.52 |
| 39 | Lilly | 2.2 | F | Miniature Pinscher | DG | 0.64 | 0.66 | 0.60 | 0.68 |
| 40 | Lola | 3.5 | F | Border Collie - Mix | - | 0.54 | 0.58 | 0.36 | 0.76 |
| 41 | Luke | 9.8 | M* | Border Collie | - | 0.52 | 0.48 | 0.44 | 0.76 |
| 42 | Luna | 2.8 | F | Siberian Husky | - | 0.49 | 0.36 | 0.32 | 0.88 |
| 43 | Luna2 | 6.4 | F* | Chihuahua | DG | 0.63 | 0.64 | 0.36 | 0.84 |
| 44 | Mago | 10.9 | M | Golden Retriever | - | 0.36 | 0.24 | 0.36 | 0.64 |
| 45 | Melissa | 13.5 | F* | German Shepherd | - | 0.51 | 0.50 | 0.48 | 0.45 |
| 46 | Michel | 10.5 | M* | Mixed Breed | - | 0.51 | 0.42 | 0.52 | 0.64 |
| 47 | Monty | 5.4 | M* | Border Collie | DG | 0.49 | 0.34 | 0.32 | 0.76 |
| 48 | Mowgli | 2.3 | M* | Labrador - Mix | DG | 0.46 | 0.40 | 0.44 | 0.68 |
| 49 | Nash | 10.4 | M | German Shepherd | - | 0.51 | 0.52 | 0.20 | 0.80 |
| 50 | Nina^x^ | 10.5 | F | Dalmatian | BUZ,DG,RL | 0.51 | 0.50 | 0.48 | 0.56 |
| 51 | Noah | 0.9 | M | Border Collie | - | 0.44 | 0.34 | 0.32 | 0.72 |
| 52 | Pipe^x^ | 0.9 | M* | Retriever - Mix | BUZ,DG | 0.39 | 0.36 | 0.24 | 0.64 |
| 53 | Pippilotta | 9.7 | F* | Irish Terrier | - | 0.49 | 0.46 | 0.20 | 0.64 |
| 54 | Poquita | 6.5 | F* | Galgo Espagnol | - | 0.43 | 0.32 | 0.24 | 0.68 |
| 55 | Quismo | 7.8 | M* | Border Collie | - | 0.51 | 0.48 | 0.40 | 0.84 |
| 56 | Raico | 4.8 | M | Standard Poodle | DG | 0.61 | 0.44 | 0.68 | 0.84 |
| 57 | Roxy | 4.6 | F | Malinois | DG | 0.47 | 0.42 | 0.44 | 0.68 |
| 58 | Schnee | 0.8 | M* | Australian Shepherd - Mix | DG | 0.47 | 0.38 | 0.20 | 0.76 |
| 59 | Sheila | 3.5 | F | Border Collie - Mix | DG | 0.59 | 0.56 | 0.52 | 0.84 |
| 60 | Sokrates | 9.5 | M* | Bardino-Mix | - | 0.38 | 0.26 | 0.44 | 0.52 |
| 61 | Stella | 2.8 | F* | Australian Shepherd | DG | 0.53 | 0.42 | 0.56 | 0.68 |
| 62 | Talie | 4.3 | M | Siberian Husky | - | 0.54 | 0.46 | 0.52 | 0.68 |
| 63 | Tika | 8.6 | F* | Husky-Mix | - | 0.41 | 0.42 | 0.36 | 0.52 |
| 64 | Todi | 12.1 | M | Mixed Breed | - | 0.39 | 0.30 | 0.44 | 0.56 |
| 65 | Ultimo | 6.0 | M | Border Collie | - | 0.50 | 0.42 | 0.36 | 1.00 |
| 66 | Ziva | 4.8 | F* | Border Collie | - | 0.69 | 0.66 | 0.60 | 0.76 |
| 67 | Zuri | 4.6 | F* | Rhodesian Ridgeback | DG | 0.44 | 0.42 | 0.44 | 0.56 |

* castrated; ^x^ excluded due to insufficient number of completed tests; BUZ = Buzzer Test, DG = Delay of Gratification Test, MC = Middle Cup Test, RL = Reversal Learning Test

**Methods**

***Delay of Gratification***

*Food preference test*

Preference tests were run before testing to ensure that dogs clearly preferred one reward type. Owners were asked which food their dogs would eat but did not have a specific preference for (low value reward (LVR); e.g. cornflakes, carrot, dry food), and which rewards their dog preferred over everything else (high value reward (HVR); e.g. cheese, sausage). To validate the owners’ suggestions, we first made sure that the dogs would eat the LVR, and if they did we presented dogs with the two food types in a repeated two-choice test. The owner was instructed to sit on a chair and to keep the dog on a short leash in front of him/her. The experimenter kneeled in front of the dog (1.2m distance), and visibly baited two plastic lids (10cm diameter) with one piece of reward per lid. The lids were differently coloured (black and white) to facilitate reward discrimination (bowl colours were counterbalanced across dogs and randomly assigned). The experimenter lifted the baited lids and leaned forward to let the dog sniff the content of both lids while the owner restraint the dog from taking the rewards. After the dog had sniffed the content of both, the lids were placed on the ground (60-70 cm from dog and 50 cm distance between lids), and as soon as the experimenter moved her hands back to her lap, the dog was released. Only one choice was allowed and as soon as the dog had approached and eaten from one lid, the other was covered and moved back again. After the dog had eaten the reward, the owner called the dog back and the next trial started. 12 trials with alternating rewarded sides were conducted, and in order to consider dogs as choosing the HVR significantly more than chance level, we set the criterion to 9 HVR-choices (one-tailed binomial: *p* < 0.02). If a dog did not reach criterion within 2 sessions other food types were chosen as rewards.
